# Advanced Diffusion MRI in the Differential Diagnosis of Hemorrhagic Cavernous Malformations in the Brain: A Case Report

**DOI:** 10.7759/cureus.69095

**Published:** 2024-09-10

**Authors:** Arda Colakoglu, Barış Genç, Kerim Aslan, Lütfi Incesu

**Affiliations:** 1 Radiology, Faculty of Medicine, Ondokuz Mayıs University, Samsun, TUR

**Keywords:** cavernoma, • diffusion tensor imaging (dti), diffusion tensor tractography (dtt), hemorrhaged cavernoma, mri, noddi

## Abstract

Cavernous malformations, low-flow vascular lesions predominantly found in the brain and occasionally in the spinal cord, can be congenital or acquired, with acquired forms often being single and congenital ones multiple. While many patients remain asymptomatic, hemorrhage from these lesions can lead to significant neurological symptoms. This case report describes a 35-year-old female with a hemorrhagic lesion in the right precentral gyrus, initially challenging to diagnose due to atypical imaging findings. Advanced magnetic resonance imaging (MRI) techniques, including dynamic susceptibility contrast perfusion and advanced diffusion methods, were employed to distinguish the lesion from other hemorrhagic conditions and suggested that the lesion is benign in nature. Follow-up MRI revealed regression of the hemorrhage and typical characteristics of a cavernous malformation. The case underscores the utility of advanced diffusion MRI techniques in differentiating hemorrhagic cavernous malformations from other pathologies, particularly in complex cases where conventional imaging may fall short.

## Introduction

Cavernous malformations, also known as cavernoma, are low-flow vascular lesions that can occur in the brain and, less commonly, the spinal cord. They can be congenital or acquired [1,2]. It is seen in approximately 0.5% of the population [3]. Acquired ones are usually single while congenital ones are often multiple [4]. They are usually located supratentorial [5]. Cavernous malformations are dynamic lesions that may show changes such as growth, shrinkage, and bleeding during follow-up [1]. Histologically, numerous dilated, thin-walled vascular structures or vascular spaces without brain parenchyma in between are seen in the lesion [6]. Hemosiderosis due to hemorrhage can be seen in macrophage and glial cells around the lesion [7]. Patients are often asymptomatic; however, hemorrhage of the lesion may cause symptoms and is an important cause of morbidity and mortality. Such symptoms are seizures because of iron’s epileptic effect, headache, vertigo, and neurologic deficits [8].

Modern diffusion imaging techniques like diffusion tensor imaging (DTI) provide more information about the brain's microarchitecture by determining the diffusion direction of water molecules, compared to standard diffusion imaging, and allow for the evaluation of the anatomy of white matter tracts. Fractional anisotropy (FA) and mean diffusivity (MD) are metrics obtained with DTI and are used in clinical practice for various pathologies. It Is also possible to acquire tractography images from DTI, which allows for the analysis of white matter tracts. On tractography images, high-grade glial tumors tend to infiltrate and disrupt white matter tracts, whereas other tumors, such as metastases, tend to push the tracts away instead of infiltrating. Diffusion kurtosis imaging (DKI) extends beyond DTI by including kurtosis metrics to capture non-Gaussian diffusion. This way it gives better insight into tissue microstructure and underlying pathologies. Mean kurtosis (MK) is the primary metric reflecting the complexity and heterogeneity of the tissue microstructure [9,10]. On the other hand, neurite orientation dispersion and density imaging (NODDI) models the brain's microstructure by differentiating between intracellular, extracellular, and cerebrospinal fluid compartments, enabling precise mapping of neurite density and orientation. This method is particularly valuable in assessing neural integrity and complexity. In NODDI, the metric neurite density index (NDI) shows us the density of dendrites and axons, while free water fraction (FWF) provides information about the free water content in brain tissue, such as edema or cerebrospinal fluid. The literature has shown that these metrics, especially those of DKI and NODDI, are beneficial in staging neoplastic diseases and various other conditions [9-12].

Nowadays, due to the prevalence of magnetic resonance imaging (MRI), we can frequently see and recognize these lesions. However, if the lesion bleeds, typical imaging findings of cavernous malformation may be masked, and it may not be possible to differentiate the lesion from other hemorrhagic lesions. Under these circumstances, advanced diffusion MRI techniques and advanced imaging methods such as DSC perfusion may contribute to the differential diagnosis. In this case, we made the differential diagnosis of the patient with a hemorrhagic lesion located in the motor cortex using advanced diffusion techniques, and when the hemorrhagic component regressed in the follow-up examination, we diagnosed the patient with cavernous malformation. We aim to highlight the contribution of advanced diffusion techniques to the differential diagnosis of hemorrhagic cavernous malformation through this case report.

## Case presentation

**Figure 1:**
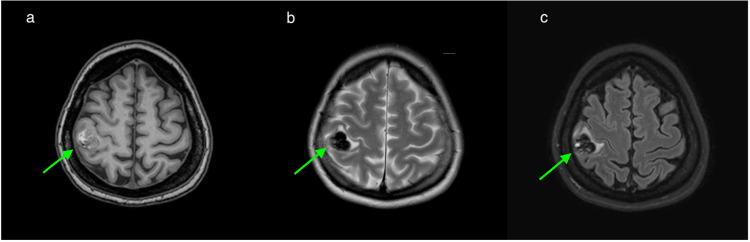
T1WI, T2WI and FLAIR images The lesion is heterogeneously hyperintense on T1WI (a). On T2WI (b) and FLAIR (c) images, the lesion is hypointense except for a high signal component in the superolateral part of the lesion. Perilesional edema is seen on T2WI and FLAIR images. T1WI : T1-weighted images; T2WI: T2-weighted images; FLAIR: fluid-attenuated inversion recovery.

A 35-year-old female without significant medical history presented to our hospital with complaints of headache and numbness in her left arm. MRI showed a hemorrhagic mass lesion accompanied by edema in the right precentral gyrus. On T2-weighted images (T2WI) and susceptibility-weighted images (SWI), the lesion was largely signal void, but a millimetric-sized high signal component was seen on its superolateral side. The lesion appeared heterogenous hyperintense on T1-weighted images (T1WI) due to blood products (Figure 1).

**Figure 2:**
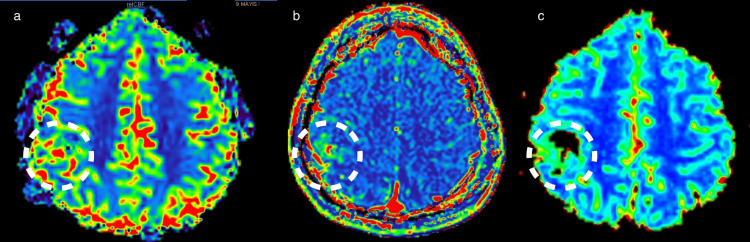
MRI T2 perfusion CBF, T1 perfusion fractional plasma volume and maximum relative enhancement images On the T2 perfusion CBF map (a), some parts of the lesion have elevated relative CBF values by up to four times. On T1 perfusion images, the lesion exhibits increased fractional plasma volume (b) and maximum relative enhancement values (c). CBF:  Cerebral blood flow.

There was no apparent enhancement of the lesion in the post-contrast images. However, in T1 and T2 perfusion MRI, an increase of perfusion parameters, up to 4 times in relative cerebral blood volume and relative cerebral blood flow (CBF), was noted in some parts of the lesion (Figure 2).

**Figure 3:**
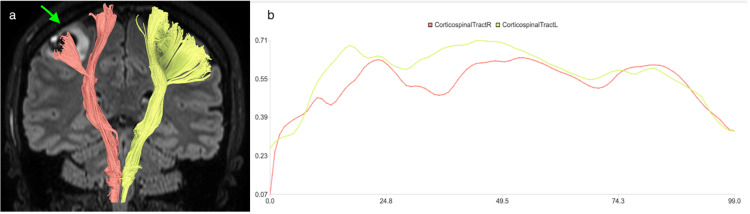
MRI tractography images and fractional anisotropy graph In the tractography images of both corticospinal tracts overlaid on the coronal FLAIR sequence (a), the lesion is seen displacing the fibers on the right side. When the fractional anisotropy graph of both corticospinal tracts (b) is evaluated along the tract, a decrease in anisotropy values is observed on the right side at the level of the lesion and inferior to the lesion, compared to the left side. FLAIR: Fluid-attenuated inversion recovery.

In addition, there was linear enhancement in the post-contrast images that was suspicious for the developmental venous anomaly (DVA) in the inferior and lateral neighborhood of the lesion. The lesion was pushing the corticospinal tract fibers on DTI. The FA value was 0.57 on the left, while it was 0.52 and decreased on the right side. Additionally, upon evaluating the tract anisotropy graph of both corticospinal tracts, a decrease in anisotropy values is noted on the right side at and below the lesion level compared to the left side (Figure 3).

**Figure 4:**
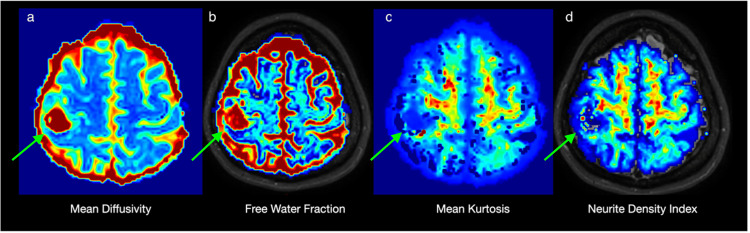
MRI advanced diffusion techniques When the lesion was evaluated with advanced diffusion techniques, an increase in MD (a) and a decrease in MK (c) were observed. An increase in FWF (b) was noted in and around the lesion. No increase in NDI (d) indicative of the presence of axons or dendrites was seen within the lesion. MD: Mean diffusivity; MK: mean kurtosis; FWF: free water fraction; NDI: neurite density index.

Advanced diffusion MRI techniques revealed an increase in MD, a decrease in MK, an increase in FWF, and a decrease in NDI in the lesion. In FWF images, water increase was also observed in the T2 high signal areas around the lesion, which is in favor of edema rather than peritumoral infiltration (Figure 4).

Furthermore, the absence of neurite within the lesion in NDI and the adjacent corticospinal tracts being pushed in DTI shows that the lesion is not infiltrative and moves us away from the diagnosis of a high-grade glial tumor.

Considering all these findings, despite the partial increase in perfusion, increased MD, decreased MK, and increased FWF both within the lesion and in the perilesional area suggest that the hemorrhagic lesion is benign in nature. We thought that the increase in CBF on T2 perfusion MRI could be related to signal loss due to blood products in the lesion. Therefore, we recommended a follow-up MRI scan two months later, and surgery was not recommended immediately.

**Figure 5:**
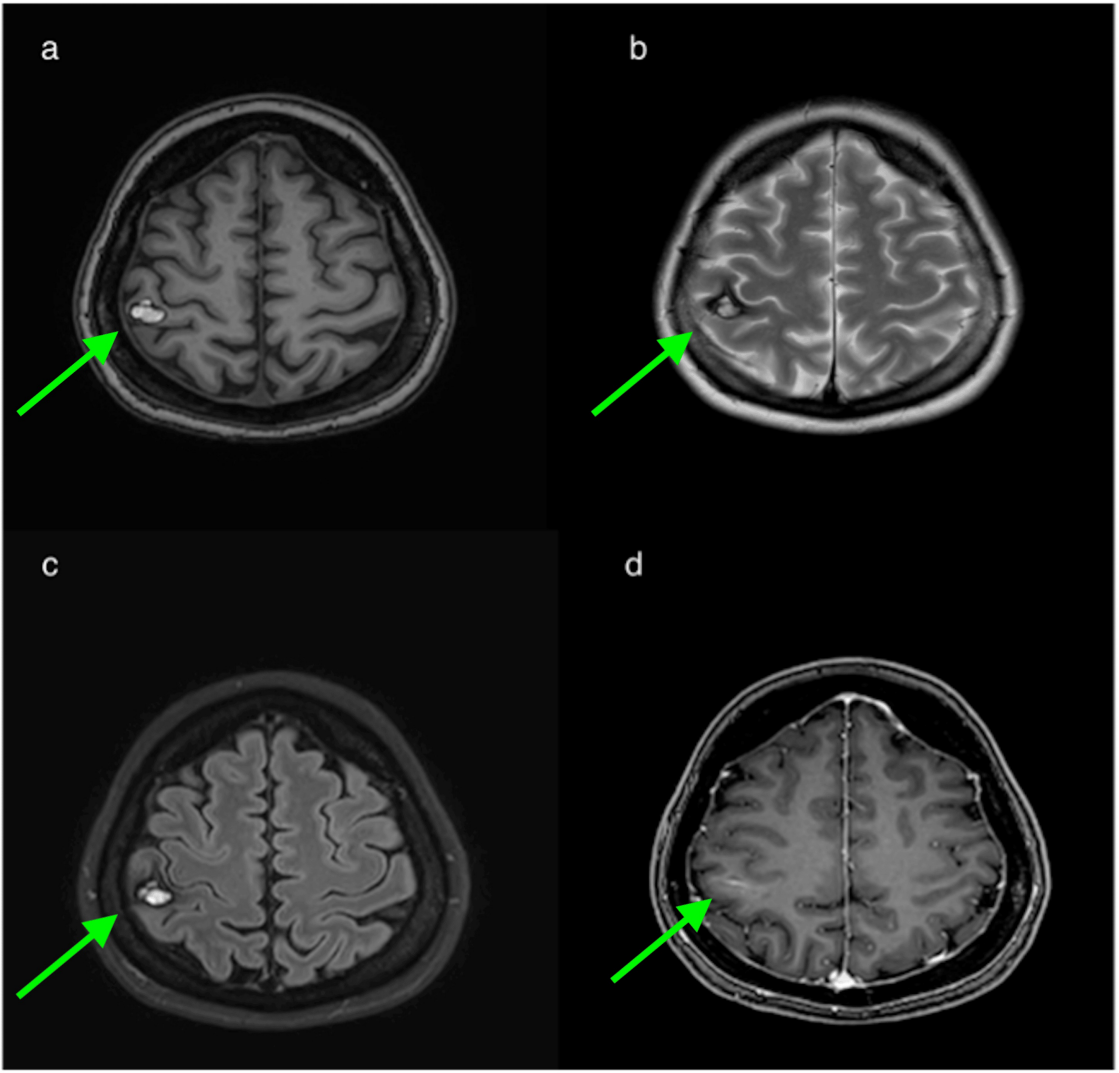
Follow-up MRI images On follow-up MRI images taken two months later, the center of the lesion is hyperintense on T1WI (a), T2WI (b), and FLAIR (c) sequences, with a low-signal hemosiderin rim on the periphery in T2WI and FLAIR. Additionally, a developmental venous anomaly running immediately inferior to the lesion is visible on the contrast enhanced T1WI sequence (d). T1WI : T1-weighted images; T2WI: T2-weighted images; FLAIR: fluid-attenuated inversion recovery.

The follow-up MRI scan showed that the dimensions of the lesion and the perilesional edema had regressed. A high signal in the center and a low-signal hemosiderin rim in the periphery of the lesion on T2WI and SWI images, along with its popcorn-like appearance, confirmed the diagnosis of a hemorrhaged cavernous malformation (Figure 5). Additionally, the follow-up examination revealed the associated DVA more clearly.

## Discussion

Cavernous malformation pathogenesis is not clearly understood. Potential causes of cavernous malformations include radiation, genetic factors, vascular malformations, head trauma, viral infections, hormonal factors, other intracranial lesions, and previous intracranial surgeries. However, there are cases in the literature with no clear etiology [2]. In one-third of cavernous malformation cases, an associated DVA is present. In these cases, it is thought that the DVA contributes to the formation of the cavernous malformation [13].

MRI is currently preferred for imaging, as computed tomography is less effective in visualizing cavernous malformations. On MRI, T2WI shows a popcorn-like shape of the lesion and a low-signal rim due to hemosiderin deposits in the periphery, with a blooming artifact visible on SWI [3,13]. The central area of the lesion can exhibit varying signal properties on T1WI and T2WI due to the presence of different aged blood products [13]. A high-signal halo around the lesion may be present on T1WI, which can help differentiate a cavernous malformation from other hemorrhagic lesions [3,13,14]. However, this finding was not present in our case. SWI performs better than other sequences for detecting small cavernous malformations [13]. Cavernous malformations generally do not enhance with contrast, but they may occasionally show variable levels of enhancement. Contrast-enhanced images can also reveal an associated DVA [1].

Due to their typical appearance, uncomplicated cavernous malformations are easily recognized on MRI scans. However, in cases of acute or recent bleeding, as in our case, the typical findings of a cavernous malformation can be masked, making it difficult to distinguish from other causes of bleeding such as neoplasms, vascular pathologies, or arteriovenous malformations. In these cases, there can be a spherical lesion consistent with acute-subacute hematoma, perilesional edema, and mass effect accompanying the typical findings of a cavernous malformation. Recurrent hemorrhage, hemosiderin rim, encapsulation, and an associated DVA support the diagnosis of a cavernous malformation in the differential diagnosis. The presence of perilesional edema may indicate that the bleeding is in the acute phase, while a high T1 signal within the lesion may suggest that the bleeding is in the subacute phase [13].

The differential diagnosis of intracranial hemorrhagic lesions includes a wide range of pathologies, such as hemorrhagic metastasis, intraparenchymal hemorrhage, vascular malformations, intralesional hemorrhage from lesions like cavernous malformations, and primary brain neoplasms. In this case, the patient had a hemorrhagic lesion located in the right motor cortex, which made obtaining a biopsy difficult. For this lesion, a definitive diagnosis could not be made using conventional MRI sequences. However, despite the partial increase in perfusion, increased MD, decreased MK, and increased FWF both within the lesion and in the perilesional area suggest that the hemorrhagic lesion is benign in nature [9-12]. We thought that the increase in CBF on T2 perfusion MRI could be related to signal loss due to blood products in the lesion. For this reason, a follow-up MRI was recommended to monitor hematoma resorption. This approach allowed us to non-invasively determine the correct diagnosis for the patient. To our knowledge, while tractography and DTI studies have been used in hemorrhagic cavernous lesions, our case is among the first to utilize NODDI and DKI methods. In this case, we demonstrated the contribution of advanced diffusion imaging to the differential diagnosis in the presence of a hemorrhagic lesion.

## Conclusions

In the case we presented, we demonstrated that although this lesion is commonly encountered in daily practice and is generally not difficult to diagnose, if it becomes complicated, distinguishing it from other hemorrhagic lesions such as metastases may become challenging on conventional sequences. In such cases, a multiparametric approach that includes both perfusion MRI and advanced diffusion MRI techniques can contribute to the differential diagnosis. Therefore, advanced MRI techniques should be utilized for hemorrhagic lesions.
